# Advances in Regenerative Sports Medicine Research

**DOI:** 10.3389/fbioe.2022.908751

**Published:** 2022-05-13

**Authors:** Liren Wang, Jia Jiang, Hai Lin, Tonghe Zhu, Jiangyu Cai, Wei Su, Jiebo Chen, Junjie Xu, Yamin Li, Jing Wang, Kai Zhang, Jinzhong Zhao

**Affiliations:** ^1^ Department of Sports Medicine, Shanghai Jiao Tong University Affiliated Sixth People’s Hospital, Shanghai, China; ^2^ Regenerative Sports Medicine and Translational Youth Science and Technology Innovation Workroom, Shanghai Jiao Tong University School of Medicine, Shanghai, China; ^3^ Regenerative Sports Medicine Lab of the Institute of Microsurgery on Extremities, Shanghai Jiao Tong University Affiliated Sixth People’ Hospital, Shanghai, China; ^4^ National Engineering Research Center for Biomaterials, Sichuan University, Chengdu, China; ^5^ School of Chemistry and Chemical Engineering, Shanghai Engineering Research Center of Pharmaceutical Intelligent Equipment, Shanghai Frontiers Science Research Center for Druggability of Cardiovascular Non-Coding RNA, Institute for Frontier Medical Technology, Shanghai University of Engineering Science, Shanghai, China

**Keywords:** regenerative medicine, sports medicine, meniscus, rotator cuff, cartilage, tendon-to-bone, bone

## Abstract

Regenerative sports medicine aims to address sports and aging-related conditions in the locomotor system using techniques that induce tissue regeneration. It also involves the treatment of meniscus and ligament injuries in the knee, Achilles’ tendon ruptures, rotator cuff tears, and cartilage and bone defects in various joints, as well as the regeneration of tendon–bone and cartilage–bone interfaces. There has been considerable progress in this field in recent years, resulting in promising steps toward the development of improved treatments as well as the identification of conundrums that require further targeted research. In this review the regeneration techniques currently considered optimal for each area of regenerative sports medicine have been reviewed and the time required for feasible clinical translation has been assessed. This review also provides insights into the direction of future efforts to minimize the gap between basic research and clinical applications.

## Introduction

**FIGURE 1:**
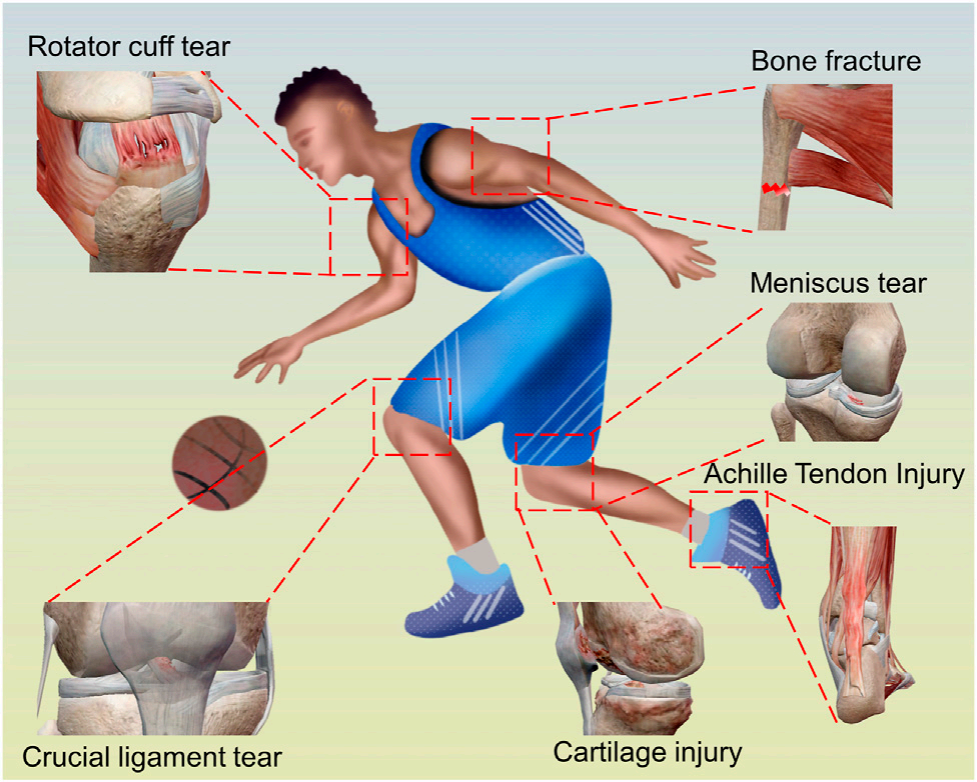
Sports medicine related injuries.

Regenerative medicine utilizes innovative approaches to explore and develop materials that can be used to replace, repair, improve, or reproduce tissues and organs in the human body (Brody, 2016). Sports medicine focuses on aspects of physical health, including the treatment and prevention of exercise-related injuries and aging-related problems that hinder the function of the locomotor system (Figure 1) (Baby, 2000; Foster, 2015; Kweon, et al., 2019). In the clinical practice of sports medicine, the prevention and treatment of conditions consequently depend on the structural and functional restoration of various related tissues and structures in the locomotor system. When structural integrity cannot be restored through repair, approaches that induce tissue regeneration become necessary, to prevent or delay the use of non-organic structures such as artificial joints. Thus, we explore the field of regenerative sports medicine, which is defined as a science that focuses on the restoration of the structural and functional integrity of the locomotor system, using techniques that induce the regeneration of tissue structures or organs. The approaches currently used in regenerative sports medicine include the utilization of organic and non-organic materials at various structural levels. From a clinical perspective, regenerative sports medicine deals with sports and aging-related conditions in different parts of the locomotor system, such as the menisci, ligaments, tendons, cartilages, and bones.

A meniscus tear is a common knee disorder (Abrams, et al., 2013) that is irreparable in many cases, and consequently, must be addressed *via* structural restoration with a partial or total meniscectomy to restore its function. Previously, allograft and synthetic menisci have been used with unfavorable or inconsistent clinical results, and thus meniscus regeneration strategies are desirable; however, they require further investigation and development (Southworth, et al., 2020; Li, et al., 2021; Veronesi, et al., 2021). Knee ligament injuries often result in ligament deficiency and joint instability and necessitate ligament reconstruction (Pike, et al., 2019). To prevent the donor site morbidity that can occur with autografts, the high failure rate with allografts, and the non-graft-bone healing associated with non-receiver transformable synthetic ligaments, receiver transformable regenerative ligaments are considered an alternative choice (Wang C. et al., 2021). Chronic Achilles’ tendon rupture often results in tendon defects that make the direct opposition of the separated tendon ends impossible and graft bridging a necessary choice; consequently, there is demand for regenerative or receiver transformable artificial Achilles’ tendons (Arshad, et al., 2021). Rotator cuff tears are mainly an aging-related condition and quite often irreparable; thus, rotator cuff grafts are required to repair defects and restore the native force chain (Novi, et al., 2018). Though various graft choices are available (Sunwoo and Murrell, 2020), receiver transformable artificial rotator cuff patches are currently considered the ideal option. Sports-related cartilage injuries and osteoarthritis are the main conditions addressed in clinical sports medicine. Clinical approaches such as micro-fractures and autogenous osteochondral graft transplantations are the main strategies used to treat small cartilage defects (Beck, et al., 2016; Guo, et al., 2018), while for large cartilage defects, cartilage regeneration is required. Bone defects, fractures, or osteotomies, osteoporosis, and osteonecrosis in the locomotor system, such as glenoid and humeral head bone defects with shoulder dislocations, glenoid bone absorption with severe shoulder osteoarthritis (OA), bone defects with high tibial osteotomy, and tibial plateau depression fractures with knee dislocations, may require bone structure regeneration (Xie C. et al., 2021). In tendon or ligament-to-bone repair, the most important goal is to restore a normal tendon–bone connection with an important fibrocartilage layer (Tits and Ruffoni, 2021). However, after soft tissue-to-bone repair the cartilage layer has been found to reappear inconsistently, which makes tendon–bone interface regeneration a critical issue (Patel, et al., 2018).

In general, regenerative sports medicine has high clinical requirements. In recent years, there has been a large amount of research in this field leading to promising outcomes. In this review, we have assessed the recent progress and assessed the time required for feasible clinical translation of the new techniques and products.

## Research Progress in Specific Fields of Regenerative Sports Medicine

### Meniscus Regeneration

#### Meniscus Physiology and the Hurdles in Regeneration

The menisci are the semilunar and wedge-shaped fibrocartilaginous tissues between the articular cartilage of the femur and tibia plateau. They have complex 3D structures to absorb shock and distribute its load through collagen fibers which are specifically aligned in a circumferential pattern. The unique zonal phenotypes in the meniscus are histologically and physiologically characterized by two distinct regions: the avascular inner zone (white–white zone), which mainly consists of glycosaminoglycan and type-II collagen with a rounded chondrocyte-like cellular phenotype, and the vascular outer zone (red–red zone), which predominantly contains higher type-I collagen with an elongated ligament-like cellular phenotype. Moreover, these two regions are separated by a middle region (red–white zone), which is a mixture of the inner and outer zones. The limited vascularity of the meniscus indicates a poor healing ability, especially in the avascular white–white zone. Thus, the major hurdles in meniscus regeneration include the inability to replicate its native anisotropic zonal structure and hence, its specialized mechanical function. Moreover, avascularity due to its unique structural properties, and the tibiofemoral articular environment that hinders the healing potency both mechanically and biochemically, have made it challenging for biomedical scientists to create matched engineering constructs for meniscus regeneration.

#### Surgical Techniques for Meniscus Regeneration

Partial lesions or defects in the meniscus reduce its propensity to heal spontaneously because of mechanical stimuli from the tibiofemoral motions and the avascularity in the white–white zone, leading to degeneration over time. Advances in techniques and tissue engineering strategies have enabled researchers to attempt to repair or regenerate these meniscal defects. Some biological promotion techniques are recommended in clinical scenarios to augment meniscus repair, such as the introduction of bone marrow stem cells using marrow venting techniques, the exogenous addition of fibrin clot, and the stimulation of adjacent healthy meniscus and synovium (Taylor and Rodeo, 2013; Dean, et al., 2017; Kwon, et al., 2019). Notably, concurrent anterior cruciate ligament (ACL) reconstruction that used bone tunnel to release the cells and growth factors from the bone marrow, has been proven to enhance meniscal repair (Chahla, et al., 2017; Dean, et al., 2017; Westermann, et al., 2017; Tagliero, et al., 2018; DePhillipo, et al., 2019). Moreover, partial meniscus replacements offer promising approaches to treat patients with segmental meniscus defects. Both collagen meniscus implants (CMI) from the USA and polyurethane polymeric implants (Actifit) from Europe have been shown to improve clinical outcomes and substantially relieve pain in patients with meniscus defects in both medium- and long-term follow-ups (van Tienen, et al., 2009; Bulgheroni, et al., 2015).

#### Tissue Engineering Strategies for Meniscus Regeneration

**FIGURE 2:**
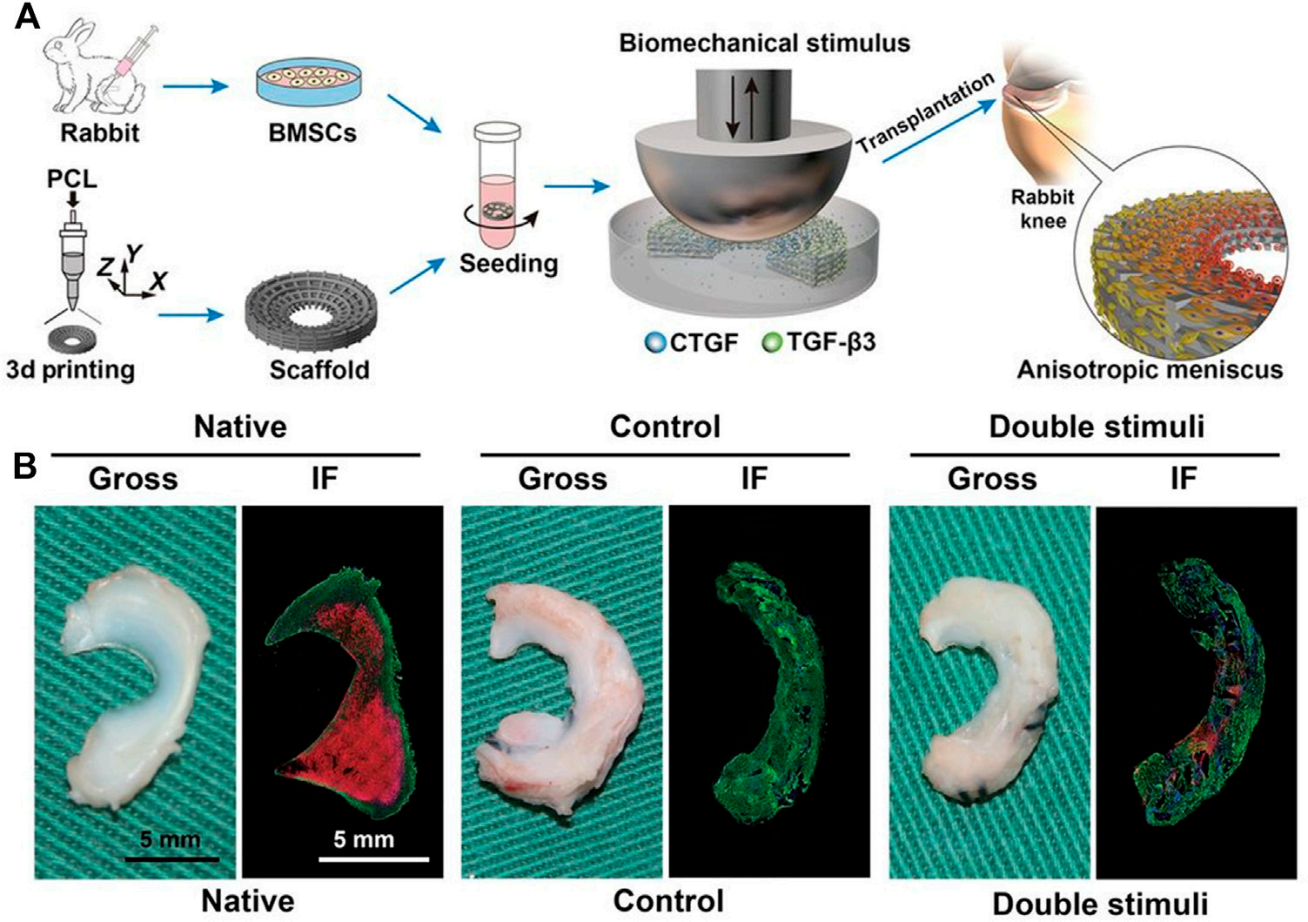
Orchestrated biomechanical, structural, and biochemical stimuli for engineering anisotropic meniscus. **(A)** Schematic diagrams for reconstruction of functional anisotropic meniscus; **(B)** Gross view and low-magnification immunofluorescence (IF) images of native or regenerated menisci at 24 weeks after *in vivo* implantation in rabbit knees. Green, COL-1; red, COL-2. Copyright 2019 American Association for the Advancement of Science.

Current surgical techniques have failed to promote meniscus regeneration, while many natural or synthetic materials, such as decellularized extracellular matrices, alginate, hyaluronan, polylactides, polyglycolides, and silk have been successfully utilized as scaffolds for meniscus engineering (Makris, et al., 2011). Among these scaffolds, decellularized extracellular matrices derived from the white–white and red–red regions of the meniscus have been shown to promote the differentiation of MSCs toward fibroblastic and fibrochondrocyte phenotypes (Shimomura, et al., 2017). Other types include injectable hydrogels that can be used to address structural defects due to their ability to form structural adaptations (Athanasiou, et al., 2013; Liu, et al., 2017). While scaffolds are beneficial due to their ability to incorporate growth factors and their initial mechanical stability, they also indicate recapitulation of the mechanical and biochemical architecture of the native meniscus, with matched stiffness and ingredient gradients (Higashioka, et al., 2014; Steele, et al., 2014; Zhu, et al., 2018; Zitnay, et al., 2018). Notably, when engineering menisci for regeneration, although an obvious choice for cell source might be fibrochondrocytes, regeneration effects are best when fibrochondrocytes are cocultured with other cell subsets (Hadidi, et al., 2016; Koh, et al., 2017; Son and Levenston, 2017; Sasaki, et al., 2018; Xie, et al., 2018). Zhang C. H. et al. (2018) used a pre-mechanically stimulated poly (ε-caprolactone) (PCL) scaffold, cocultured with rabbit bone marrow stem cells, for meniscus replacement and found that the pretreated scaffold was a better choice for inducing tissue regeneration (Figure 2).

#### Current Clinical Studies and the Challenges in Clinically Translating Meniscus Regeneration

Currently, a few scaffolds for meniscus engineering are undergoing clinical trials with a focus on cell-based therapies. Cell Bandage, a collagen sponge embedded with autologous bone marrow-derived MSCs, was applied in clinical practice to close the torn edges and defects of the meniscus, and is assumed to potentially promote healing. In another clinical trial in humans, a chondrogenic composed of allogeneic bone marrow-derived MSCs was administered to the knee, and reported to effectively decrease visual analog scale pain scores. While meniscus repair products for clinical applications are currently lacking, preliminary outcomes suggest that cell-based therapies are a positive and promising road ahead; however, they have also identified challenges that must be overcome. In the clinical translation of engineered meniscus products, an insufficient source of autologous cells is the primary issue. The use of non-articular cells, however, seems to be a potential strategy to alleviate the scarcity of cells for autologous meniscus therapies (Makris, et al., 2011; Utomo, et al., 2016). Additionally, the high-quality autologous neo-tissues required to consistently regenerate the meniscus are difficult to obtain, as demonstrated by the large biological variability observed between donors (Martin, et al., 2017; Vapniarsky, et al., 2018; Kwon, et al., 2019). Therefore, well-characterized allogeneic tissues and cell banks should be established to enable suitable neo-tissues to be provided stably and avoid disease transmission, and this is likely to solve the intractable problem of biological variability. Furthermore, mechanical biomimicry when engineering the meniscus should be achieved, as the native meniscus allows for frictionless tibiofemoral joint movement and load distribution, which may be related to positive long-term healing outcomes (Elder and Athanasiou, 2009; MacBarb, et al., 2013; Huwe, et al., 2018). If the meniscus can be successfully generated after overcoming the aforementioned challenges, then the avascular white–white zone of the meniscus leads to difficulties in both implant protection and its integration into existing native tissues (Arvayo, et al., 2018; Vapniarsky, et al., 2018). For surgeons and biomechanical researchers, developing appropriate techniques and protocols to enhance the vascular supply to implants should be a priority (Vapniarsky, et al., 2018; Kwon, et al., 2019). In addition to vascularity, the engineering meniscus must also adjust to the inflammatory microenvironment, especially in an injured or diseased joint with its complex biochemical conditions. Therefore, modifications by decellularization and antigen removal when engineering the meniscus are required to minimize the immunoreaction of xenogeneic or allogeneic menisci to ensure implant survival and integration. Li et al*.* (2021) fabricated silk/graphene oxide-based meniscus scaffolds, which consisted of tannic acid and Sr^2+^. The scaffold exerts anti-inflammatory and reactive oxygen species elimination effects, which protect against cartilage degeneration and delay OA development after meniscus injury.

The promising progression which will ultimately lead to the application of tissue-engineered therapies for meniscus regeneration in clinical practice, is evident in current clinical trials. In the near future, tissue engineering strategies may rapidly emerge for the development of meniscus regeneration products, which could potentially provide long-term solutions for patients.

### Cruciate Ligament Regeneration

#### Common Strategies for Cruciate Ligament Regeneration

**FIGURE 3:**
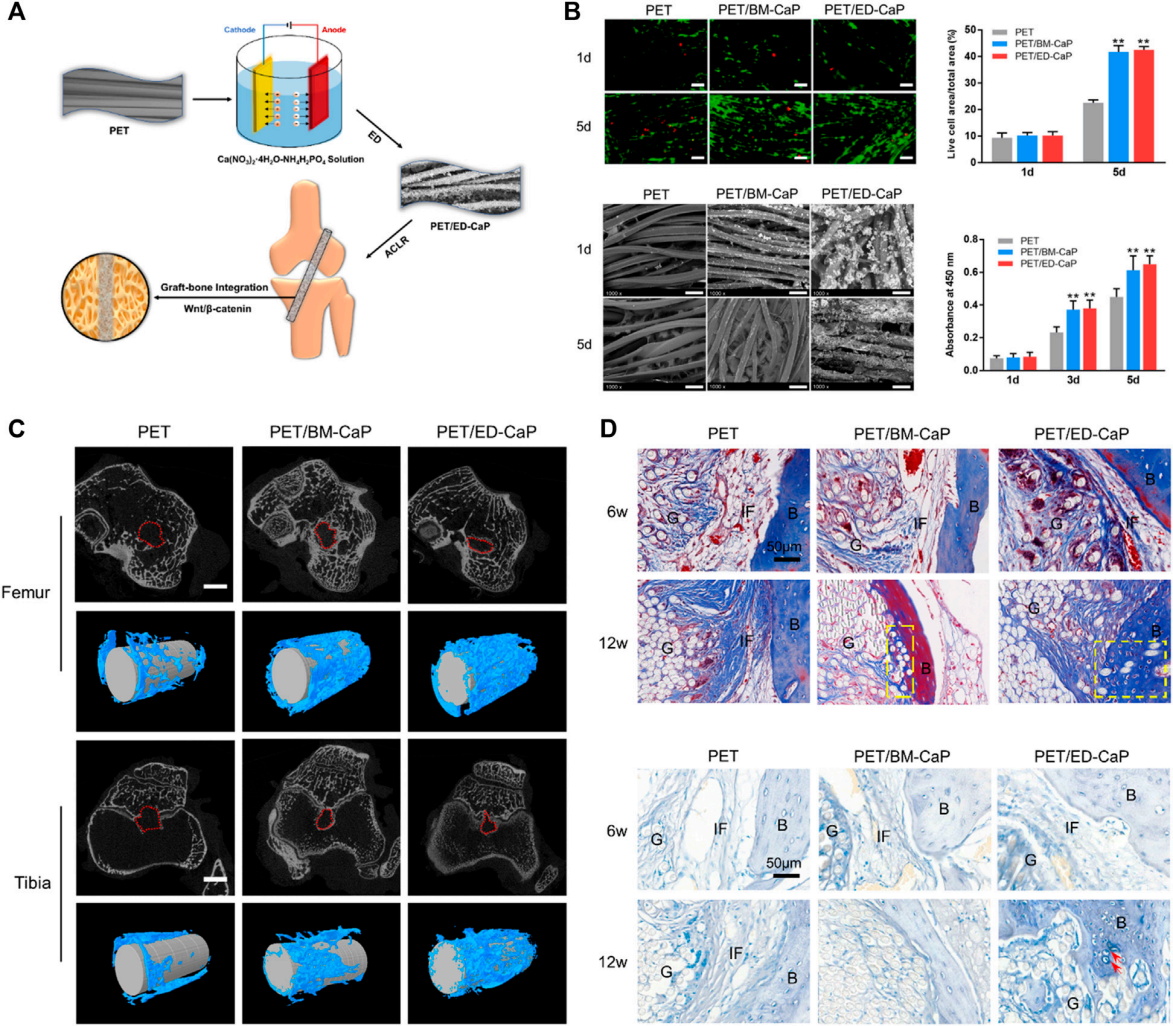
Electrodeposition of calcium phosphate onto polyethylene terephthalate artificial ligament enhances graft-bone integration after anterior cruciate ligament reconstruction. **(A)** Electrodeposition of calcium phosphate onto polyethylene terephthalate artificial ligament; **(B)** The viability and SEM morphology of MC3T3-E1 in the PET, PET/BM-CaP and PET/ED-CaP groups; **(C)** Micro-CT analysis of the PET, PET/BM-CaP and PET/ED-CaP groups at 12 weeks after surgery; **(D)** Masson and toluidine blue staining results of pathological sections in the PET, PET/BM-CaP and PET/ED-CaP groups at 6 and 12 weeks after surgery. Copyright 2021 Elsevier.

Knee crucial ligament injuries are common in sports medicine, and often occur during adolescence and young adulthood (Petersonand Krabak, 2014). Ligament reconstruction is the main solution to prevent subsequent cartilage and meniscus damage, thus improving quality of life (Mastrokalos, et al., 2005; Petersonand Krabak, 2014). Clinically, autografts and allografts are the two most common graft types used for surgical ligament reconstruction (Cai J. Y. et al., 2021). However, donor site morbidity remains an inevitable problem associated with their use (Yilgor, et al., 2012), and allografts carry additional risks of disease transmission, infection, rejection, low availability and quality, and high failure rates (Jackson, et al., 1993). Xenografts [porcine bone-patellar tendon–bone (BTB)] were harvested by Galili et al. and treated with recombinant alpha-galactosidase and glutaraldehyde for ACL reconstruction (Galili and Stone, 2021). The authors have completed preclinical trials with monkeys and progressed to clinical trials, and they have reported no significant differences in the functional performances of the porcine BTB group and cadaveric allograft group at the 24 months follow up, if the missing/contaminated cases were excluded (Stone, et al., 2007; Van Der Merwe, et al., 2020). The potential advantages of the xenografts are that they could help address the quality concerns and availability problems that occur with allografts. However, like allografts, xenografts also have the disadvantages including the possibility for disease transmission, infection, and rejection. Moreover, the process of utilizing an animal originated graft with human tissue, namely graft “humanization,” is difficult and will require further investigation (Van Der Merwe, et al., 2020). To address this, artificial ligaments have been developed in recent years. To date, those ligaments that are clinically available, have been made of non-degradable materials or non-receiver transformable materials, such as polyethylene terephthalate (PET) and polyethylene, which are characterized by their hydrophobic properties and inferior biocompatibility and lead to poor graft-host bone healing after implantation (Figure 3) (Ai, et al., 2017; Cai J. et al., 2021). The development of receiver transformable artificial ligaments is another scope and direction for future ligament research.

#### Artificial Materials for Cruciate Ligament Regeneration

Teuschl *et al.* (Teuschl, et al., 2016) fabricated novel degradable silk fiber-based artificial ligaments and used biological materials, biodegradable polymers, and composite materials in ligament fabrication for ACL reconstruction in a sheep model. The silk ligaments could induce new tissue ingrowth and stimulate ACL regeneration *in vivo*. However, the balance between the degradation rate of the materials and the regeneration and remodeling rate of the tissues was not controllable. Furthermore, it is unknown whether the regenerated tissues could maintain the function of the knee at a level similar to that of the native ACL, as functional recovery and a return to sports cannot be fully evaluated in quadruped animal models.

The combined use of receiver transformable and non-transformable materials is an additional option. Mengsteab *et al.* (Mengsteab, et al., 2020) incorporated PET fibers into the poly (L-lactic) acid (PLLA) bioengineered ACL matrix to fabricate a PET/PLLA hybrid ligament. The hybrid ligament demonstrated great peak loads and promoted the regeneration of ACL in a rabbit model.

#### Future Perspectives

Despite these encouraging results, further work is required to optimize the properties of newly developed grafts for crucial ligament reconstruction of the knee. We believe that decellularized scaffolds with ready-made collagen and degradable artificial ligaments are the two most promising graft types for ligament reconstruction in future clinical practice. However, prior to application, issues regarding ligament development must be addressed, as it is vital that the host-graph response be regulated and controlled. Moreover, there is a need to explore the regenerative competent microenvironment, which is the articular cavity that can induce tissue ingrowth into the graft. For clinical use, the functional assessment of the knee is more important than the regeneration and healing assessment, as the regenerated or remodeled ligament should be able to mimic the function of the native ligament.

### Achilles Tendon Regeneration

#### Surgical Techniques for Achilles Tendon Repair

End-to-end repair of the chronic Achilles tendon is appropriate when the gap is 2 cm or less, while the V-Y technique, turndown flaps, autograft tendon transfer, and reconstructions using allograft, xenogeneic, or synthetic biomaterials are required for larger defects with or without the preservation of the paratenon (Kraeutler, et al., 2017; Maffulli, et al., 2018; Muller, et al., 2018; Chen and Hunt, 2019). Regardless of the multiple surgical management strategies, the ideal treatment for tendon injury is to promote Achilles tendon regeneration after gap formation (Sun, et al., 2018).

#### Strategies for Achilles Tendon Repair

**FIGURE 4:**
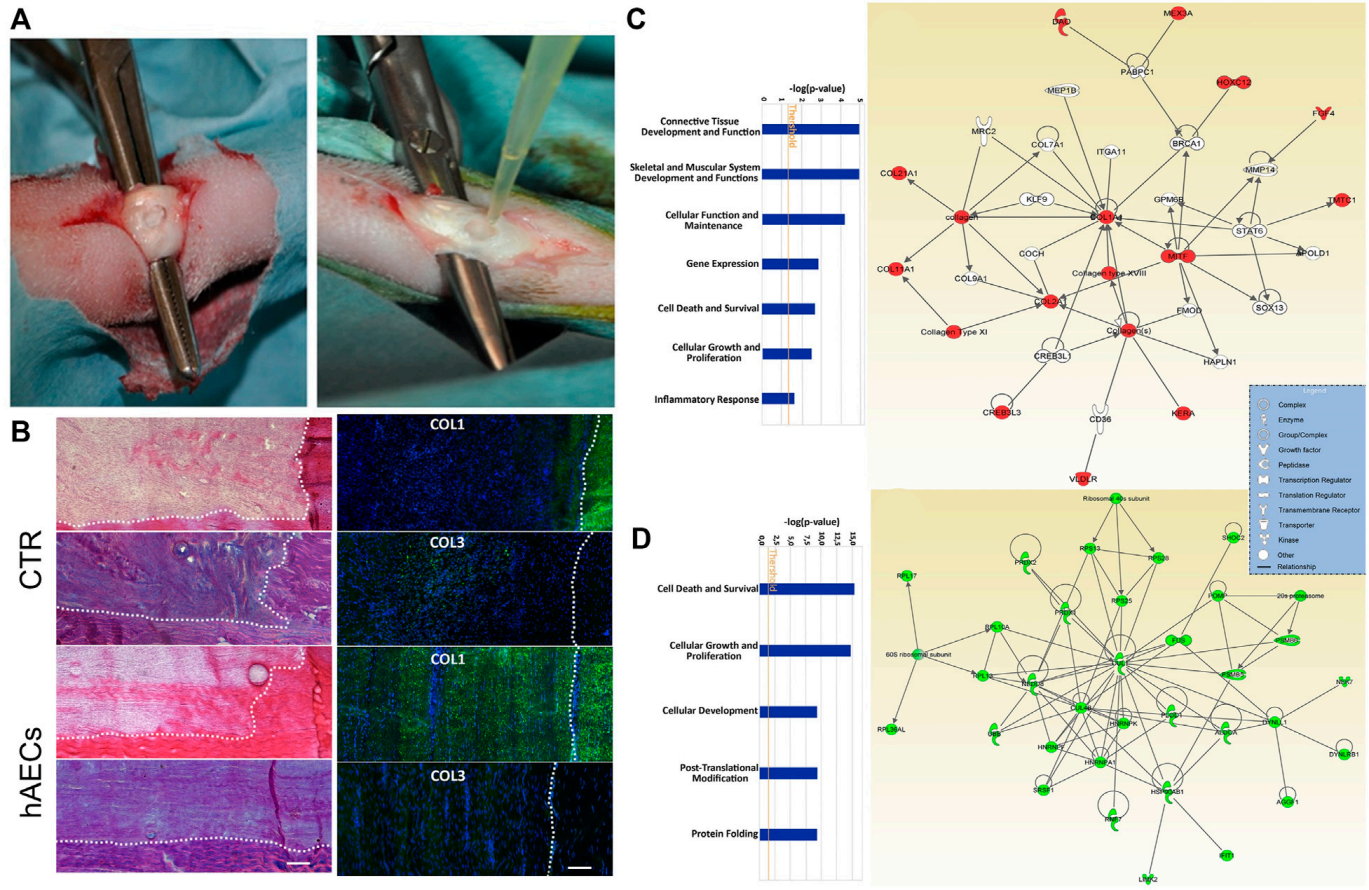
Therapeutic potential of hAECs for early Achilles tendon defect repair through regeneration. **(A)** Circular defects of 5 mm created in the Achilles tendons. One defect was filled with fibrin glue, whereas the contralateral with 10 × 106 PKH26-stained cells suspended in fibrin glue (bottom); **(B)** Representative haematoxylin–eosin-, Herovici and immunofluorescent staining of CTR (control) and human amniotic epithelial cell (hAEC)-treated tendons. **(C)** Key functions associated with genes found to be up-regulated in hAECs and the top-scored network; **(D)** Key functions associated with genes found to be down-regulated in hAECs and the top-scored network. Copyright 2017 Wiley.

The literature on Achilles tendon regeneration is limited mostly to laboratory studies using porcine small intestinal submucosa (Badylak, et al., 1995), acellular tendon matrix (Gungormus, et al., 2015; Zhang C. H. et al., 2018), and collagen (Sun, et al., 2018) or collagen gel (Shen, et al., 2010) as scaffolds. Moreover, exogenous cell transplantations such as for tenocytes (Gungormus, et al., 2015) and human amniotic epithelial cells (Barboni, et al., 2018) have been applied but restricted by the cell source, immune rejection, ethics, and injured microenvironment (Figure 4) (Harris, et al., 2004; Sun, et al., 2018). Chemokines like SDF-1α and recombinant SDF-1α containing a collagen-binding domain (CBD) have also been reported to promote endogenous tendon regeneration by inducing extracellular matrix production and avoiding the above drawbacks of exogenous cell transplantation in clinical applications (Shen, et al., 2010; Sun, et al., 2018). The local application of combined ascorbic acid and T_3_ also showed the potential benefits for accelerated tendon healing (Oliva, et al., 2019). Platelet-rich fibrin (PRF), with the delivery of cytokine and growth factor, induced more organized collagen fibers *in vivo* and promoted tenocyte viability and tenogenic phenotypic differentiation *in vitro* (Wong, et al., 2020). Similarly, platelet-rich plasma (PRP) is widely used and has been proven to be effective for tendon healing *in vivo* (Chiou, et al., 2015). However, Zhang et al. (Zhang C. H. et al., 2018) found that the combined PRP was no better at repair-augmenting effects than the scaffolds alone for Achilles tendon regeneration.

#### Future Perspectives

Promising laboratory findings reported in the literature suggest that there will be important implications for the practical application and clinical translation of tendon regeneration (Cai C. et al., 2021; Cai et al., 2022; Wang et al., 2022). However, few clinical studies have been reported to date, and most are case reports and small case series (Chen and Hunt, 2019) with a few translational animal models (dog, horse, *etc*.) (Sun, et al., 2018; Badylak, et al., 1995; Gungormus, et al., 2015; Zhang C. H. et al., 2018; Shen, et al., 2010). Additionally, therapeutic perspectives are not achievable until the critical challenges relating to the scaffold, cell, or chemokine use (source, induction condition, genomic compatibility, dose, *etc*.) are solved. The combination of scaffold implanted with cell or chemokines, however, is encouraging for future studies and promising for human clinics.

### Rotator Cuff Regeneration

#### Treatment of Rotator Cuff Repairs Is an Ongoing Challenge

Rotator cuff defects are the main issues that occur in shoulder repair, and the rate of surgical failure is reportedly up to 94%, especially for large and massive tears after simple repairs (McElvany, et al., 2015; Lewington, et al., 2017; Saveh-Shemshaki, et al., 2019). Various scaffolds have been used to replace the native tissue structures in rotator cuff repairs. Most scaffolds consist chiefly of extracellular matrix and chemical polymer, which provides a bridge for connecting tendon and bone tissues, and adsorbs the fibroblast secreted collagen matrix (Guevara, et al., 2020). These scaffolds have been utilized in rotator cuff tendon tissue engineering for several decades (Steinhaus, et al., 2016; Zhao, et al., 2017). They usually combine bioactive substances to promote rotator cuff regeneration, such as stem cells and growth factors. There are currently three types of rotator cuff tendon scaffold used: xeno-patches, allo-matrices, and synthetic films (Steinhaus, et al., 2016; Coons and Alan, 2006; Wang D. et al., 2021). However, imperfect tissue regeneration is an ongoing problem.

#### Xeno-Patches for Rotator Cuff Repair

Xeno-patches extracted from extracellular matrices are effective bioactive scaffolds for tendon engineering and can be applied in surgical implantations to rotator cuff defects (McGovern, et al., 2018). The acellular xenografts derived from the porcine dermis and small intestine were used in a large animal model for infraspinatus repair to evaluate the effects of tendon regeneration. Nicholson et al. (Nicholson, et al., 2007) found that intestinal and porcine dermis patches were almost replaced by tendon-like tissues at 24-week, but a foreign body reaction was observed in the conjunction site of the tendon and xenograft. Ultimately, the cause of failure was the same for the dermal and intestinal groups. The potential immune reaction and associated chronic foreign body responses were the main concerns. This reaction may result from the residual DNA in the Xeno-tissue, even if processed by decellularization. Another problem is the hyper acute rejection caused by α-Gal. The α-Gal epitope exists in non-primate mammals (Naso, et al., 2011; Platts-Mills, et al., 2021), and therefore, the epitope antibody is produced in humans, which specifically binds to xeno-tissue.

#### Allo-Matrices for Rotator Cuff Repair

**FIGURE 5:**
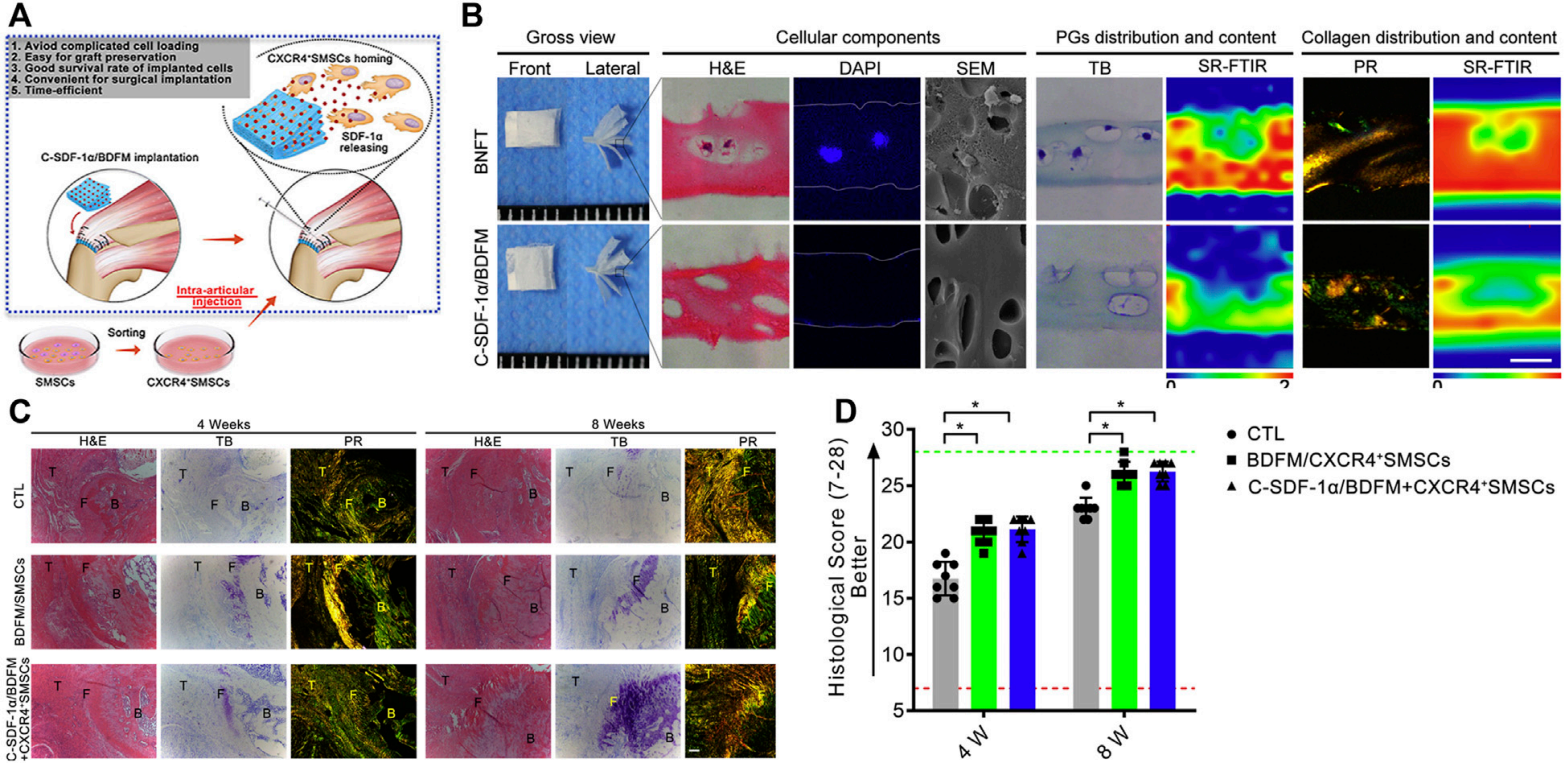
Functional decellularized fibrocartilaginous matrix graft for rotator cuff enthesis regeneration: A novel technique to avoid *in-vitro* loading of cells. **(A)** Developing a cell-free graft with chemotaxis to recruit postoperative injected cells; **(B)** Macroscopic observation, histological analysis, and synchrotron radiation-Fourier transform infrared spectroscopy analysis of the book-type nature fibrocartilage tissues and C-SDF-1α/BDFM, sections stained with hematoxylin and eosin (H&E), DAPI, toluidine blue (TB), and picrosirius red (PR); **(C**,**D)** Histological analyses of regenerated fibrocartilage during RC healing. Copyright 2020 Elsevier.

Allo-matrices originated from decellularized cadaveric human tissues and were found to have the capability to bridge tendon tissue defects, with a low risk of tissue-scaffold rejection (Fini, et al., 2012). A study by Adams et al. (Adams, et al., 2006) explored the histological and biomechanical processes of allo-matrices, by utilizing allo-dermal matrices to bridge tendon and bone in an animal model for infraspinatus repair. The fibroblast infiltration and new collagen deposition were surrounded by dermal matrices 6 weeks after implantation, and at 24 weeks, a more mature tendon-like tissue was formed in the allo-dermal group. The biomechanical properties of the regenerated tissues were promising. However, there were only small-scale clinical trials conducted to evaluate their performance (Zhao, et al., 2017). Even though no serious allo-matrix related complications were observed, and clinical outcomes appeared to be good, some potential problems still existed. Similar to the xeno-patches, there were concerns about the residual DNA. The residual DNA may cause immune inflammatory reactions and increase the proliferation of the scaring tissue (Lewington, et al., 2017). There is also evidence that the mechanical properties of the allogenic matrices are decreased when compared with that of the auto-tendons. To better induce tendon–bone interface regeneration, Chen et al. (2019) added recombinant SDF-1α to the decellularized bone–fibrocartilage–tendon composite and injected synovium-derived mesenchymal stem cells (SMSCs) into the repair site. They found that the fabricated scaffold was better at recruiting the SMSCs and resulted in well regeneration of the tendon–bone interface 8 weeks after surgery. However, the option proposed in this study is too complicated for clinical application. Neither the addition of recombinant SDF-1α nor the injection of stem cells has been approved by the administration (Figure 5).

#### Synthetic Polymers for Rotator Cuff Repair

Owing to the ongoing focus on immune reactions for both the xeno-patches and allo-matrices, synthetic polymers that are of great interest for tendon regeneration engineering have been identified (Qiu, et al., 2021; Zhao, et al., 2021). Degradable polymers, including PLLA, poly-dioxanone (PDO), and poly (lactic-co-glycolic) acid (PLGA) have been used as supports to create multifunctional scaffolds. These synthetic films consisting of regularly arranged fibers exhibit stronger mechanical characteristics than the scaffolds derived from bio-tissues (Gachon and Mesquida, 2021). The controllable arrangement plays a role in cell migration, with fibroblasts well aligned with the axis of the polymer fibers when compared to the un-aligned films in a random orientation. The polymer film had fewer immune responses when compared with that of the xeno-and allo-patches, indicating that it could be a potential scaffold to bridge the tendon gap. Yokoya et al. (Yokoya, et al., 2012) used the PLGA sheet to repair the full-thickness defect of the rotator cuff in a rabbit model and observed that a greater proportion of type-I collagen was generated in the PLGA sheet with mesenchymal stem cells. The repaired site also had a better ultimate strength when compared with that of the controls without mesenchymal stem cells. Studies that have explored synthetic films have achieved encouraging results, but the products of the polymer film degradation were identified as a concern (Silva, et al., 2020). High levels of chemical composite have a toxic effect on fibroblast proliferation and inhibit collagen deposition. These toxic effects vary with different polymers, and therefore, more research is required to ensure that the degradation product levels are safe.

#### Future Perspectives

These bio-scaffolds are assumed to offer ideal structural binding sites for tissue integration. Synthetic films have the advantage of mechanical properties and low rates of immune reaction. Several scaffolds have emerged in recent years; however, they have not yet been used in routine clinical surgery (Zurina, et al., 2020). The ideal scaffold should be able to meet the mechanical strength of the cuff tendon and provide bioactive binding sites that promote fibroblast-mediated healing and tendon regeneration.

### Cartilage and Osteochondral Regeneration

#### Simple Cartilage Regeneration

The common types of joint bone defects include partial cartilage injury, full-thickness cartilage injury, and osteochondral defect (Chow, et al., 2004; Shkhyan, et al., 2018; Kato, et al., 2019). The repair and regeneration of damaged cartilage tissue is one of the most challenging problems in the field of tissue engineering and regenerative medicine.

Until now, promoting cartilage regeneration has been one of the greatest difficulties in the field of regenerative medicine, due to the limited self-healing ability of cartilage tissues (Wang, et al., 2020; Zhang, et al., 2020). Natural and synthetic substances are the two main biomaterials used to restore cartilage (Chae, et al., 2021; Qiao, et al., 2021; Schuurmans, et al., 2021). The aim is mainly to improve cell adhesion and promote the growth and dynamic migration of regenerative tissues. Scaffolds are not only regarded as physical substrates, but in the biological environment, scaffolds are related to each other through clear chemical exchanges and physical stimulation through cells and adjacent tissues. Therefore, scaffolds are mainly used to support cell culture, infiltration, proliferation, and differentiation caused by signal factors and mechanical stimulation (Gardner, et al., 2016; Lynch, et al., 2016). Scaffolds are dissimilated into categories such as nanomaterials, biomimetic materials, biological enhancers, and hydrogels, and they can be used to bind chondrocytes or can be placed at cartilage defect sites. Hua et al. (2021) fabricated multifunctional hybrid optical crosslinking (HPC) hydrogels by photopolymerization and photopolymerization of imine crosslinking. Loaded with chondrocytes, the scaffold was used for cartilage defect repairing through arthroscopy in a pig model. Six months after implantation, an ideal layer of cartilage was regenerated. However, the development of an optimal scaffold that can induce cartilage regeneration is ongoing. The other critical issue in isolated cartilage regeneration is how to increase the adherence of the regenerated cartilage to the bone underneath.

#### Scaffolds for Osteochondral Regeneration

**FIGURE 6:**
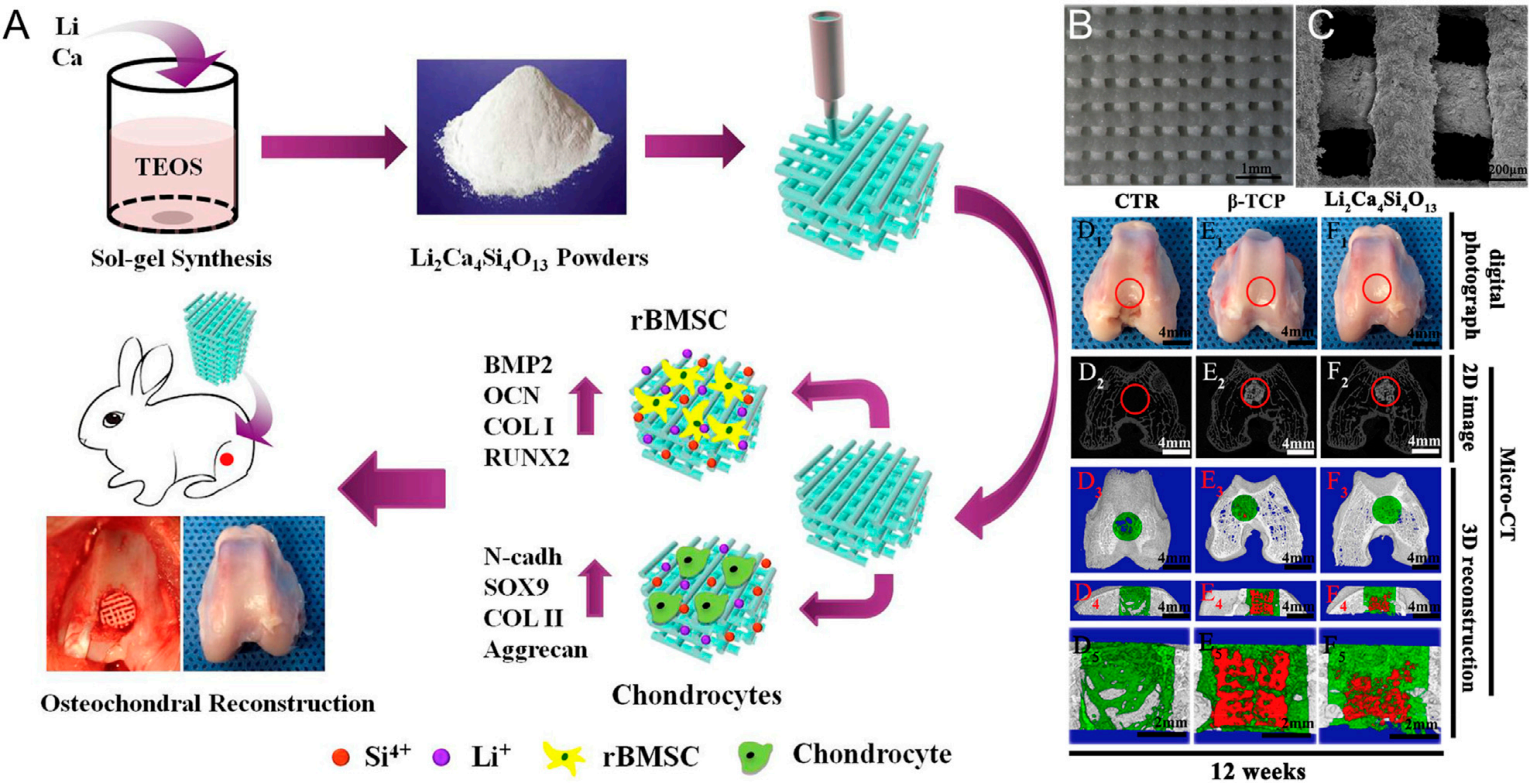
3D printing of a lithium-calcium-silicate crystal bioscaffold with dual bioactivities for osteochondral interface reconstruction. **(A)** Schematic illustration of application of Li2Ca4Si4O13 scaffolds for osteochondral reconstruction; **(B)** SEM images of 3D-printed Li_2_Ca_4_Si_4_O_13_ scaffolds after fabrication and **(C)** after soaking in the simulated body fluids for 14 days; Macro-photographs showed the defects in the control group and the other two experimental groups (**D**_**1**_: blank control without scaffolds, **E**_**1**_: pure β-TCP scaffolds, **F**_**1**_: Li_2_Ca_4_Si_4_O_13_ scaffolds) at 12 weeks of post-surgery; (D_2_ – F_2_) showed 2D projection images of the three experimental groups at week 12; (D_3_ – F_3_) showed the transverse view of 3D reconstruction images of the three experimental groups at week 12; (D_4_ – F_4_) showed the sagittal view of 3D reconstruction images of the three experimental groups at week 12. Copyright 2019 Elsevier.

A method to repair osteochondral defects, the terminal stage of a cartilage defects, is urgently required (Eldridge, et al., 2020; Hall, et al., 2021; Kim, et al., 2021). At present, serious defects can only be treated by arthroplasty (Pirosa, et al., 2021; Xie J. et al., 2021). However, the commonly used artificial joints based on non-degradable materials, such as metals and ceramics have some disadvantages, including their high cost, limited biocompatibility, foreign body rejection, and long-term loosening (Labek, et al., 2011; Valdes, et al., 2012; Sakellariou, et al., 2016). Achieve bone–cartilage composite tissue regeneration and permanent joint physiological function reconstruction remains a challenging problem. Using biomimetic scaffolds to induce *in situ* osteochondral regeneration is expected to be an important option for future joint function reconstruction (Figure 6) (Radhakrishnan, et al., 2018; Chen et al., 2019; Xu, et al., 2019; Zhu, et al., 2019). The tissue engineering of osteochondral integrated scaffolds imitates a normal osteochondral structure, as well as the natural osteochondral components in composition, to achieve a double bionics structure and components, which will eventually enable the effective repair and regeneration of osteochondral defects. However, due to the complex anatomical structure and component content of normal bone and cartilage, as well as the dynamic changes in time and space that occur in the regeneration area, the repair and regeneration of the osteochondral defect area is not just a simple “filling” of new tissue. It requires subchondral bone regeneration to support hyaline cartilage, and hyaline cartilage is closely combined with bone to generate cartilage–bone interface integration and thus the concurrent regeneration of both cartilage and bone.

To achieve a breakthrough in osteochondral regeneration, we must focus on preparing integrated bionic scaffolds that accurately simulate the microenvironment of osteochondral regeneration, and solve the following key scientific problems: *1*) accurate simulation of 3D shapes, multi gradient structures, regional specific matrix components, and the microenvironment factors of joints to prepare a bionic osteochondral scaffold (Choe, et al., 2021; Lee, et al., 2021; Liu, et al., 2021); *2*) achievement of osteochondral tissue regeneration and biological joint construction *in vitro*; and *3*) realization of the industrialization of integrated bionic stents and clinical transformations of the biological joints. The accumulation of separate tissue regeneration research for the cartilage and bone and the application of emerging cutting-edge technologies in recent years has facilitated breakthroughs regarding these technical problems.

At present, osteochondral scaffolds are commonly used in experimental research and clinical applications including natural biomaterial scaffolds, synthetic scaffolds, bioceramic scaffolds, bioactive glass, extracellular matrix scaffolds, and composite scaffolds.

The biological and mechanical properties of various tissue-engineered osteochondral scaffolds are different due to their different components and structures. For example, although natural biological scaffolds have the advantage of good biocompatibility, high cell affinity and degradability, which are conducive to adhesion and proliferation following the infiltration and recruitment of cells, they also have disadvantages, such as poor mechanical properties, rapid degradation rates, and limited sources. Synthetic scaffolds and bioceramics have good mechanical properties, strong plasticity, controllable degradation, and unrestricted wide sources. Their corresponding disadvantages are poor biocompatibility, low cell affinity, lack of hydrophilicity of some scaffolds, and their degradation products may have certain toxicities. With the development of tissue engineering, to overcome the shortcomings of single materials, two or more materials have been combined according to the principles of complementary characteristics and advantages, to design an ideal scaffold that can meet the needs of osteochondral tissue engineering.

Composite scaffolds combine the advantages of individual scaffolds, such as a controllable degradation rate, good cell compatibility, good hydrophilicity, and appropriate biomechanical strength. Su et al. (2021) prepared cartilage layers *via* collagen II and chitosan with a pore diameter of approximately 100 μm and a bone layer *via* the PLGA with a pore diameter of 500 μm. The chondrocytes labeled by nano magnetic particles were planted on this biphasic scaffold to observe their growth, proliferation, and distribution on the scaffold, to further investigate the effects of this method on the repair and regeneration of bone and cartilage. The experimental results showed that the combination of a scaffold structure and cells labeled by novel technology has good application prospects for repairing regenerated osteochondral defects.

#### Biomedical Material Fabrication in Osteochondral Regeneration

Rapid progress in biomedical material fabrication has been made in the field of osteochondral regeneration, including 3D bioprinting, electrospinning, aerogels, hydrogels, and drug loaded microspheres (Deng, et al., 2021; Gao, et al., 2021; Jiang, et al., 2021). However, there are still some challenges, such as the accuracy and stability of 3D biological printing technologies and the flexibility and function of the products. A potential clinical application of bioprinting is to develop “*in vivo* bioprinting” technology, which can accurately “print” cell materials on the damaged parts with the help of handheld print heads, to directly repair cartilage defects of different shapes and thicknesses. This technique has great potential for the development of individualized treatment plans and will help to eliminate the need for a secondary surgery.

In addition to 3D printing technologies, tissue-engineered hydrogel scaffolds have also been utilized in cartilage repair. However, it is difficult for hydrogels to meet the advantages of high porosity, good mechanical properties, toxicity, biocompatibility, and a controllable degradation cycle. Most of the hydrogels can only satisfy one or two advantages. A composite hydrogel that can synchronize the degradation rate with the regeneration rate of cartilage tissue could be promising as a repairing material for the treatment of cartilage defects.

The applicational prospects for tissue engineering electrospinning scaffolds is optimistic, with high porosity and bionic extracellular matrix structures, but there are still many problems that must be addressed, such as: *1*) electrospinning scaffolds seriously affecting the adhesion, proliferation, and differentiation of seed cells on materials; *2*) solving the contradictions between the mechanical strength and degradation rates of materials; and *3*) unclear the teratogenicity and tumorigenicity of materials in the human body.

While the tissue engineering of osteochondral integrated scaffolds can solve some of the existing problems in traditional treatments, there are also shortcomings. If there is no in-depth study on the mechanisms for the repair and regeneration of osteochondral integrated scaffolds, it is impossible to clarify the repair and regeneration mechanisms for the defect area from a microscopic cellular and molecular level. Moreover, the osteochondral integrated scaffold has double bionics for its structure and composition, which cannot be compared with the normal osteochondral structure at both biological and mechanical levels; further, special materials similar to the natural osteochondral structure-cannot be found. In addition, the calcified layer and tidal line play important roles in the structure of bone and cartilage, but the integrated bionic scaffold is still difficult to completely biomimic these unique structures. Therefore, at present, the problem of the calcification of the cartilage layer and easy separation between the two layers of biphasic and multiphasic scaffolds has not yet been solved. Nevertheless, use of new preparation technologies and methods, such as 3D printing and electrospinning technologies, discovery or synthesis of new scaffold materials, and cooperation between medicine, industry, materials, biological structures, biomechanics, and integrated bionic scaffolds can finally solve the clinical scientific problem of osteochondral defects.

### Bone Regeneration

Bone defects, fractures or osteotomies, osteoporosis, and osteonecrosis in the locomotor system may require bone-structure restoration to achieve regeneration (McFarland, et al., 2016; Khira and Salama, 2017; Choi and Rhee, 2017; Zhang Y. et al., 2018). Some examples of such cases are glenoid and humeral head bone defects occurring in shoulder dislocations, glenoid bone absorption in cases of severe shoulder osteoarthritis (OA), bone defects occurring in high tibial osteotomy, and tibial plateau depression fractures in cases of knee dislocation. Bone regeneration is defined as the process wherein bone-grafting materials are replaced by newly formed bone (Wei et al., 2022). Till now, autologous grafts have been suggested as the gold standard for bone regeneration. However, the accompanying donor-site complications and the limited availability of autografts hamper their extensive use in clinical applications. Meanwhile, allogeneic bone grafts are challenged by vascularization issues and disease transmission risks (Giannoudis, et al., 2005; Laurencin, et al., 2006). Thus, there is an urgent need for the development of biomaterials in sports medicine. There are different strategies used to treat bone defects, such as *1*) simple artificial bone material, *2*) artificial bone material with bioactive factors, and *3*) artificial bone material with stem cells.

The ideal scaffold would have an appropriate hierarchical architecture that would permit normal metabolic activity as well as the migration, proliferation, and differentiation of cells together with angiogenesis and bone ingrowth. As an example, a highly porous scaffold would have a greater surface area that would promote improved osteogenic effects by allowing greater mass exchange and adsorption of growth factors (Wu, et al., 2014; Tang, et al., 2016). The pore size of the scaffold is also critical for good bone regeneration, as the presence of smaller pores leads to hypoxic conditions that promote pre-osteogenic osteochondral formation while larger interconnected pores promote directional osteogenesis (Karageorgiou and kaplan, 2005; Zhang, et al., 2013). Porous surfaces also stimulate interactions and linkages between the implant and the bone, and the pore size is critical for bone integration. For example, it was found that while 300-μm pores produced the most lamellar bone, the process of osseointegration was longer than with 200-μm pores (Xu, et al., 2011).

In this review, we have focused on research over the past 5 years into the optimization of the architectural, chemical, and surface features of bone graft substitutes (BGS) for the promotion of bone regeneration and osteointegration. Three-dimensional printing permits the creation of BGS tailored for the individual patient by optimization of both mechanical and structural characteristics. The optimizing structure permits specific correspondence between the BGS and the patient’s body, leading to more rapid postoperative recovery (Tan, et al., 2017). Although titanium is most commonly used for 3D printing, materials such as bioceramics and polymers such as polyetheretherketon (PEEK), which allow custom design, are only being investigated at the pre-clinical stage at the moment (Mustafa, et al., 2011; Li, et al., 2017a; Yang, et al., 2017). The issues that are being addressed in the use of these novel ceramic materials include optimal mechanical characteristics, architectural design, and chemical properties to enhance both porosity and degradability. Optimal surface characteristics are vital for osteogenic cell adhesion to the BGS, leading to the promotion of new bone growth (Colquhoun and Tanner, 2015; Fernandez-Yague, et al., 2015; Babaie and Bhaduri, 2018). These properties can be manipulated by coatings that promote bone regeneration.

As bone repair is a complex process that is dependent on various growth factors, we discuss the application of active biomolecules for promoting bone repair (Krishnan, et al., 2006; Carragee, et al., 2011; Polak, et al., 2011; Kim, et al., 2012; Santo, et al., 2013; Mumith, et al., 2017). These applications can be divided into three approaches are three approaches: *1*) the application of recombinant growth factors, individually or as mixtures, together with a natural or calcium phosphate matrix, such as BMP-2 (Infuse bone graft), BMP-7 (OP-1 putty), and rhPGDF-BB (Augment bone graft^®^); *2*) the use of ECM-derived peptides targeting cellular receptors, such as B2A (B2A2-K-NS) and P-15; *3*) the use of small molecules targeting pathways that influence none mass, including parathyroid hormone (PTH), Nel-like molecule-1 (NELL-1), and LIM mineralization protein-1 (LMP-1). These molecules may affect bone mass directly or indirectly by inhibiting negative modulators of bone mass and thus promoting increased bone mass.

The third approach used stem cells as part of a cell-based construct. This requires the presence of progenitor cells allowing the formation of new tissue through interaction with host cells, stimulatory factors, and support providing cells with 3D cues for new tissue formation. The progenitor cells used include bone marrow stromal cells (BMSCs), adipose-derived mesenchymal cells (ASCs) and periosteum-derived stem cells (PDSCs) (Ingber, et al., 2006; Bolander, et al., 2016; Bolander, et al., 2017). However, the necessity of pre-incubating the biomaterial with the cells complicates the engineering process considerably and also reduces the viability of the cells, leading to high production costs. In addition, the scaffolds may not have sufficient ability to promote vascularization *in vivo* and may thus be unable to maintain cell and tissue viability (Lenas, et al., 2009). Because of these issues, this approach has not proved popular in clinical practice.

**FIGURE 7:**
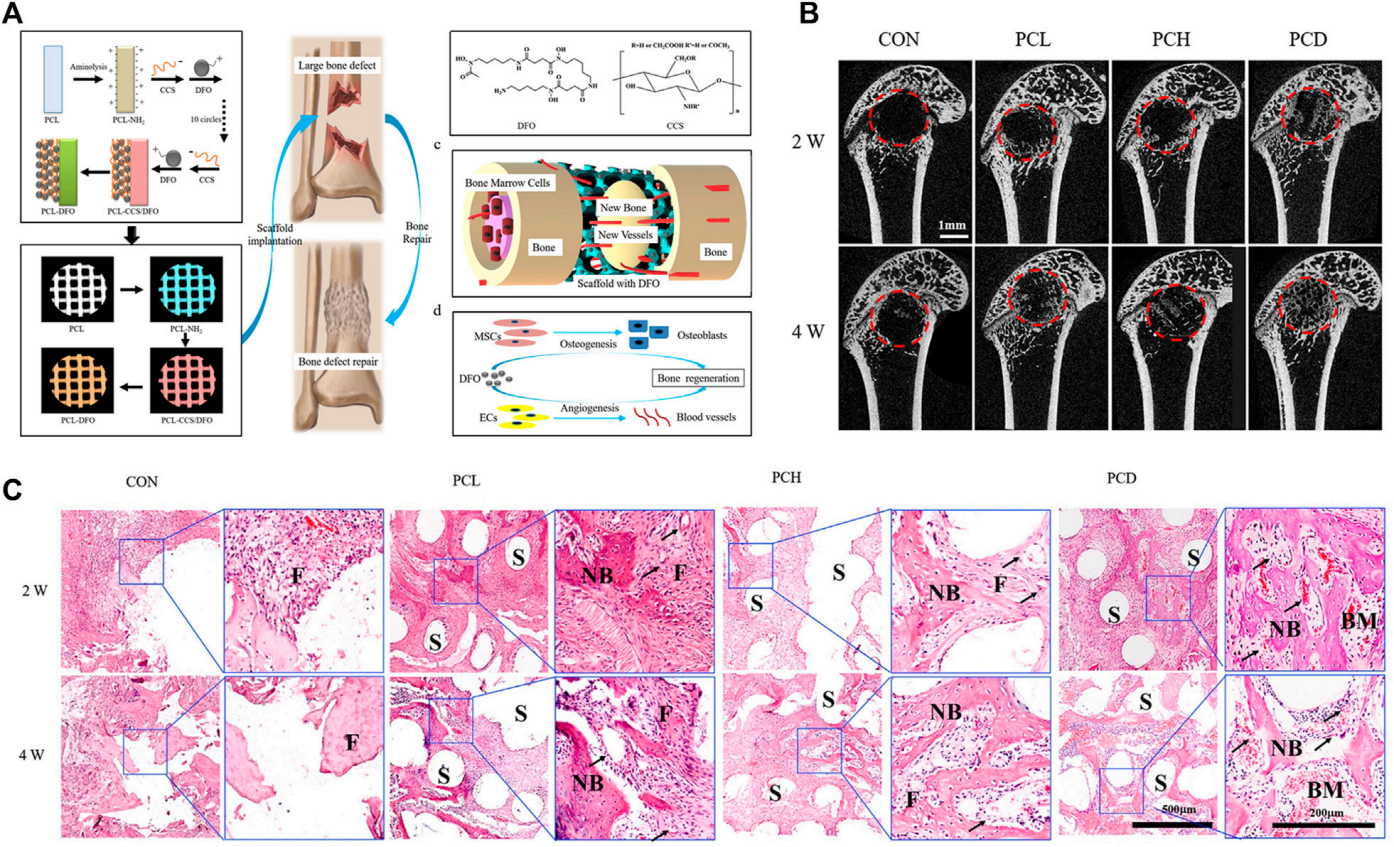
Vascularized 3D printed scaffolds for promoting bone regeneration. **(A)** Schematic diagram of bridging deferoxamine (DFO) on the surface of 3D printed polycaprolactone (PCL) scaffold and its biological function for bone regeneration in bone defect model; **(B)** Micro-CT analysis of the effect of scaffolds on bone repair *in vivo*; **(C)** Representative images of hematoxylin-eosin (HE) staining of the decalcificated bones slice, showing the new formed tissue including the fibrous tissue (F), newly mineralized bone tissue (NB) and the structure of scaffolds (S). Copyright 2019 Elsevier.

Bone tissue engineering approaches were devised to address the shortcomings of bone grafts and alloplasts and to promote the repair of bone defects and fractures. Both biological derivatives and synthetic materials have been used for scaffold fabrication, singly or in combination. The developments in the field include the use of scaffolds together with gene therapy and stimulatory factors (Farberg, et al., 2012; Pountos, et al., 2016; El Bialy, et al., 2017; Yan, et al., 2019), while recent advances in the 3D printing of scaffolds open new directions for effective bone regeneration (Ding, et al., 2013; Gong, et al., 2015; Lee, et al., 2017; Li, et al., 2018) (Figure 7). Nevertheless, many challenges remain. Recent reports have emphasized the importance of local microenvironments for the success of these scaffolds. In addition, more understanding of the precise functions of the active biomolecular constituents, such as their influence on inflammation or bone precursor cells, is required. The precise control and delivery of these molecules in the correct doses are necessary to prevent undesirable side effects. There is intensive research in the form of pre-clinical studies to understand the underlying mechanisms of these therapies and their effective applications. Translation to clinical practice also requires many regulatory steps and costs.

### Tendon–Bone Interface Regeneration

#### Poor Tendon–Bone Interface Healing After Tendon/Ligament Reconstruction

One of the main problems in the functional reconstruction of tendons and ligaments is the poor healing of the tendon–bone interface (Yang, et al., 2017; Xing, et al., 2020, Zhu, C., et al., 2021). Natural tendon–bone interfaces can be categorized into four portions: tendon, uncalcified fibrocartilage, calcified fibrocartilage, and bone (Weimin, et al., 2013; Liu, et al., 2019). The transition of the structures effectively prevents the structural damage caused by a sudden tension, by gradually distributing tension across the interface (Lu and Thomopoulos, 2013). From a mechanical perspective, this structure perfectly connects ligament and bone tissue with an elasticity modulus of 200 MPa and 20 GPa, respectively, which also increases the strength of the insertion to avoid the avulsion of the tendon. However, after injury, even with proper surgical treatments, scar tissue takes the place of the transitional structure in the tendon–bone interface, which greatly decreases its mechanical properties (Zhu, J., et al., 2021). Hence, inducing the regeneration of natural tendon–bone interfaces is a major issue when treating tendon–bone interface injuries.

#### Scaffolds Fabricated for Tendon–Bone Interface Regeneration

A decellularized extracellular matrix is a common option for tendon–bone interface regeneration, as it possesses ideal biocompatibility and a natural microstructure. Through a combination of physical, chemical, and enzymatic treatments, Su et al*.* (2019) successfully fabricated decellularized triphasic hierarchical bone–fibrocartilage–tendon composites, with the preservation of natural microstructures and mineralization. After 8 weeks implantation occurred into the tibia bone tunnel, and the fabricated decellularized bone–fibrocartilage–tendon composite induced a notably larger amount of bone regeneration in the bone tunnel when compared with that of simple decellularized tendon tissue. However, it is still unclear whether the decellularized bone–fibrocartilage–tendon composite can induce cartilage regeneration between the tendon–bone interface, which is the main issue in tendon–bone interface regeneration. Additionally, the fabrication procedure of decellularized bone–fibrocartilage–tendon composite is relatively complicated. It is quite difficult to achieve a balance between the preservation of the natural microstructure and the elimination of remnant cell debris and consequently, there is no current gold standard method for the field.

Decellularized small intestinal submucosa (SIS) has been generated for use as a commercial medical implant as numerous studies have indicated that many bioactive factors can be preserved, even after the decellularization procedure (Meng, et al., 2021; Singh, et al., 2021). However, unlike the decellularized bone–fibrocartilage–tendon composite, decellularized SIS scaffolds lack the required microstructures between the tendon–bone interface. Even though decellularized SIS has been successfully used in many other tissue repair processes, recent clinical studies jointly suggested that the use of decellularized SIS did not (Su, et al., 2021)result in better clinical results when used for rotator cuff repair surgery (Iannotti, et al., 2006; Bryant, et al., 2016). The retear rate and American Shoulder and Elbow Surgeons shoulder score were not improved, which indicated that the tendon–bone interface regeneration was not properly induced by a decellularized SIS scaffold (Sclamberg, et al., 2004).

**FIGURE 8:**
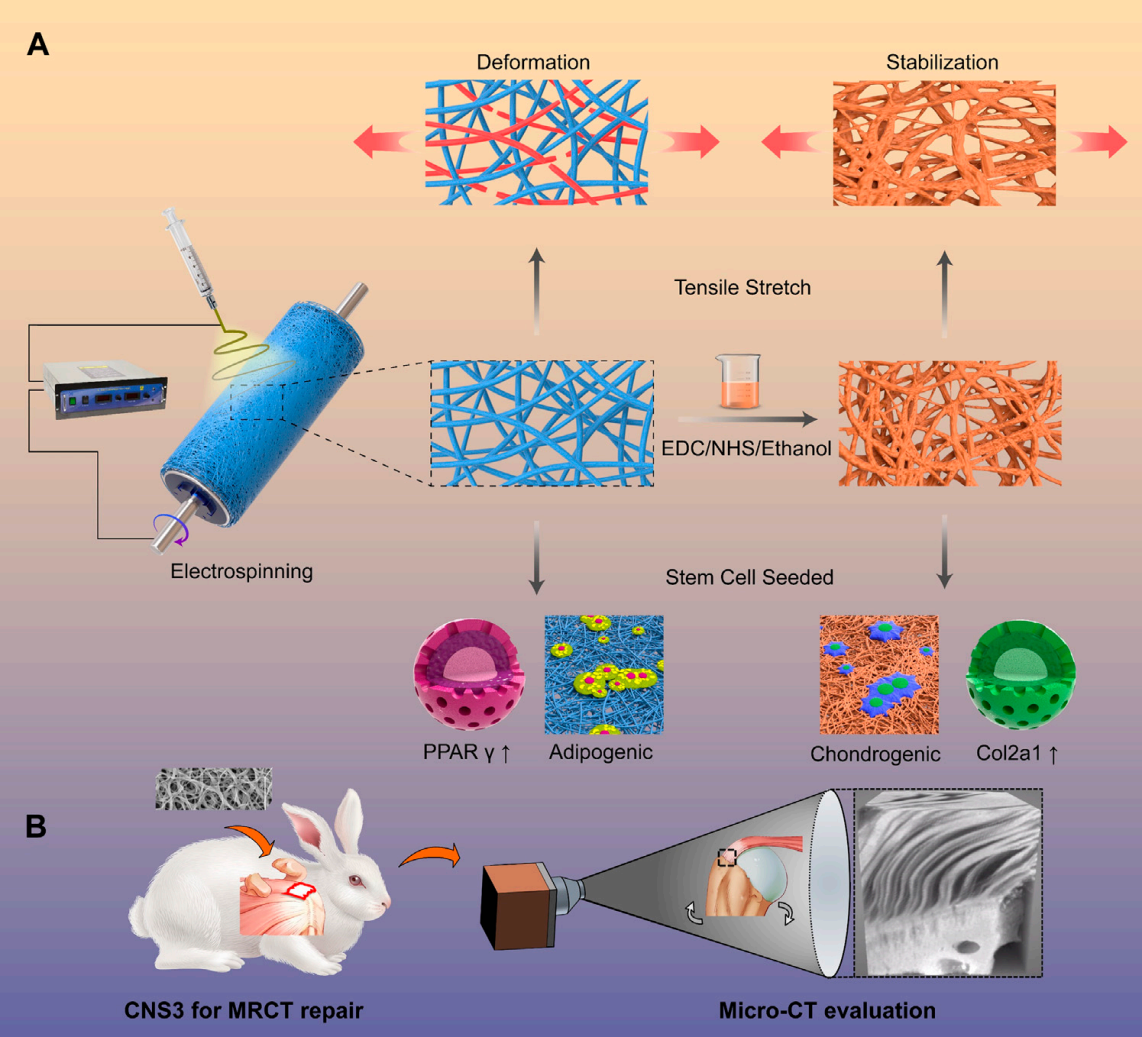
Crimped nanofiber scaffold mimicking tendon-to-bone interface for fatty-infiltrated massive rotator cuff repair. **(A)** Schemata of fabrication of the crimped nanofibrous scaffold for massive rotator cuff tear repairing; **(B)** Representative micro-computed tomography (micro-CT) images of the proximal humerus and quantitative analysis. Copyright 2022 Elsevier.

When compared with a natural extracellular matrix, artificial polymer scaffolds have advantages, including good quality control reliability and microstructure adjustability. Nevertheless, unlike a natural extracellular matrix, the artificial polymer scaffold lacks biocompatibility, biodegradability, and inducibility; highlighting the need to select appropriate materials. PLGA and PCL are common material options that have been deemed safe by the Food and Drug Administration (Li, et al., 2015). By electrospinning, the fabricated scaffold is equipped with the similar microstructure when compared with that of a natural extracellular matrix (Hua, et al., 2021). However, the microstructures of the tendon–bone interfaces are very different from the extracellular matrices of other tissues (Rossetti, et al., 2017). The orientation of fiber in the extracellular matrix of the tendon–bone interface changes from aligned-to-random, suggesting that the fabricated scaffold should also mimic the transitional microstructure differences (Deymier-Black, et al., 2015). Xie C. et al. (2021) fabricated electrospun scaffolds with aligned-to-random microstructures and morphologies of the tendon fibroblasts were cultured on the scaffold, and were organized and haphazardly oriented, respectively. In addition to the differences in the microstructures, there is also a gradient density for calcium in the tendon–bone interface. As PLGA and PCL are bioinert materials, which cannot induce bone regeneration, bioactive factors were commonly added to the scaffolds. In a previous study, we produced a PCL scaffold combined with gradient calcium phosphate silicate (CPS) content and found that the multilayer gradient composite effectively increased tendon–bone healing at the tear site, where better tissue cellularity and gradient mineralized cartilage formation were observed (Su, et al., 2021). Wang D. et al. (2021) crosslinked nanofibrous scaffolds to fabricate a scaffold, which simulates the microstructure of the natural tendon-to-bone interface, and 3 months after implantation in rabbit, it fully regenerated the unique 3D structure of the tendon-to-bone interface (Figure 8). However, the aforementioned studies were also too complicated for application in industrial production. Controlling the fiber orientation transition and gradient density of calcium is difficult using the current industry techniques.

#### Expectations in Tendon–Bone Interface Regeneration

To date, no medical implant for tendon–bone healing has been developed. Considering the difficulties in balancing the biocompatibility, biodegradability, inducibility, microstructure, and gradient density of the calcium, the time frame for a relevant implant is not short; however, it will be improved with time. Previous studies have either presented procedures that are too sophisticated or added bioactive materials not approved by the Food and Drug Administration. Scaffolds that can effectively induce tendon–bone interface healing, are easy to manufacture, and are highly reliable, are urgently required.

## Materials for Sports Medicine Injury

Autografts are recommended as the “gold standard” for most common sports medicine injuries, including ACL tears and bone defects (Cho, 1975; Clancy, et al., 1982; Howe, et al., 1991). Although autografts contribute to an increased healing rate, both structurally and functionally, donor site injury and limited tissue sources restrict their applications (Piva, et al., 2009). Allografts can not only reduce surgery time but also reduce damage and complication at the donor site. However, a long-term follow-up study indicated that, when compared with autografts, allografts could prolong the post-surgery recovery time, increase the incidence of infection, and the risk of spreading infectious diseases (Dahlstedt, et al., 1989; Good, et al., 1989; Greenberg, et al., 2010).

Natural biological materials are one of the major material sources of tissue engineering. SIS is a natural, acellular, degenerable, extracellular collagen matrix material which is mainly composed of helically interweaving type-I and -III collagen (Badylak, et al., 1989; Badylak, et al., 1995). Studies confirmed that using SI as an ACL supplementary repair could contribute to neuro-vascularization and cell growth (Nguyen, et al., 2015). However, Badylak et al. (1989) found that there was a reduced tensile resistance in the mucous membrane of the small intestine after 3 months in the experiment for goat ACL reconstruction, which could not meet the prerequisite in clinical biomechanics. Silk is a natural material and highly recommended for its desirable biocompatibility and mechanical strength (Li, et al., 2014; Teuschl, et al., 2016). However, the uncontrollable speed of silk degeneration, limited cellular affinity, and unstable mechanical strength immediately after surgery all imposed restrictions on its application (Soong and Kenyon, 1984).

Non-degradable polyester materials, such as PET, are another option as they are equipped with high mechanical strength. Ligament advanced reinforcement system (LARS) is a representative product of non-degradable artificial ligaments and it was advantageous to the patients who needed to be back to the field with a high-level performance. Considering its disadvantages, such as poor hydrophilicity, inertness, and no osteogenic active ingredient, the LARS ligament would lead to the formation of a scar at the ligament-bone interface and loss of long-term effectiveness (Li, et al., 2012; Tiefenboeck, et al., 2015). Some studies have focused on surface coatings to improve bioinert material defects. Studies have shown that coating the surface of LARS ligaments with fibroin or hydroxyapatite can effectively promote the degradation of LARS ligaments and the tendon–bone healing of inert material in the bone tunnel (Ai, et al., 2017; Cai, et al., 2018; Wang, et al., 2018). However, there are still problems with the current technique, such as nonuniform surface coating on the ligament and binding force, which greatly limits the clinical applications of the products.

Single polymer/macromolecular based scaffolds (such as polylactic acid and poly (caprolactone)) have been recently developed and are being explored for potential clinical use (Correia Pinto, et al., 2017; Erisken, et al., 2008; Zhang, et al., 2005). Such scaffolds can be equipped with relatively good mechanical properties and structural machinability which can be adjusted for different requirements. Research has indicated that they can gradually degrade in 600 days and maintain a certain degree of strength, which provides stable conditions for tissue regeneration (Blaker, et al., 2011). Most of the absorbable polymer materials have a low hydrophilicity and cellular affinity. Furthermore, polylactic acid can produce acid metabolites during the degeneration process and thus cannot form real tissue inductivity (Lu, et al., 2005; Cardwell, et al., 2014). For successful tissue engineering, the single polymer/macromolecular based scaffolds should meet several criteria (Zhao, et al., 2014a; Zhao, et al., 2014b; Zhao, et al., 2015; Li, et al., 2017b). First, good biocompatibility and mechanical properties should be confirmed *in vitro* to lay the foundation for further *in vivo* implantation. During degeneration, the initial, middle-stage, and final grafts should cooperate with induced regenerative tissues to support sufficient strength. Finally, the toxicity of the degeneration product should be strictly limited.

## Summary

There is a high demand for tissue regeneration techniques in the field of sports medicine, which make regenerative sports medicine crucial for the treatment of injuries and diseases in the locomotor system. There are several conundrums, however, that are limiting the application of these techniques (Tables 1, 2). The first issue is how to restore like for like, i.e., how to recreate tissues that mimic the native tissues in both structure and function. The second issue is how to fabricate a structure that can overcome the defects of the native structure to ensure that its function is improved and the potential for reinjury is reduced.

**TABLE 1:** Common sports medicine injuries and their promising treatment methods.

Common sports medicine injury	Clinical gold standard treatment	Shortage
Meniscus tear	Meniscectomy or meniscal repair	Progression of osteoarthritis and decreased sports function
Cruciate ligament tear	Autograft reconstruction	Decreased mechanical property of ligament
Achilles tendon tear	Surgical repair	Decreased sports function
Rotator cuff tear	Partial repair or rotator cuff reconstruction	The recurrence rate is 40–94%
Cartilage tear	Conservative treatment	Progression of osteoarthritis
Bone defect	Autograft	Limited source
Tendon-to-bone injury	None	—

**TABLE 2:** Promising treatments’ advantage and disadvantage.

Potential regeneration methods	Advantage	Disadvantage
Allograft	Ideal biocompatibility and bioactivity	Limited source and donor site injury
Xeno-patches	Wide range of sources	Risk of infection and immunological rejection
Natural materials	Biocompatibility	Limited bioactivity and mechanical property
Non-biodegradable artificial synthetic material	Mechanical property and ideal structural machinability	Structural failure in the long-term
Biodegradable artificial synthetic material	Relatively good mechanical property and Structural machinability	The balanced control of degenerative and regenerative rate; Toxicity of some degeneration product

For meniscus regeneration, tissue engineering strategies could potentially generate a meniscus like cellular or acellular structure with or without growth factors. Progress has already been made in restoring its native anisotropy and zonal organization. Further research should be conducted to understand how to restore its hooping effect from the collagen structure and the connection between the anterior and posterior horns and the tibia, as well as how to ensure its healing to the peripheral capsule ligament and how to obtain sustained vitalization of the neonatal structure to reduce the risk of reinjury.

For cruciate ligament, rotator cuff, and Achilles’ tendon regeneration, the goal is to obtain a viable type-I collagen dominated fibrous structure that can heal to the bone and soft tissue it connects. For the collagen regeneration-inducing technique that utilizes degradable synthetic material as the main construct, there is still a long way to go before it will be able to induce the regeneration of sufficient volumes of collagen. The collagen transformation-inducing technique is more practical with readily available collagen. However, vitalizing the structure in the intraarticular environment, obtaining a mature ligament or tendon structure with sufficient final strength, and achieving satisfactory ligament or tendon**–**bone healing requires further investigation.

Osteochondral regeneration induced with a biomimetic scaffold is a promising strategy to solve the problem of cartilage regeneration and cartilage–bone adhesion. Progress has been achieved in the development of a scaffold simulate native osteochondral construct with regard to its microstructure, components, and bioactive stimulator. Breakthroughs are still required, however, to obtain a scaffold that can induce bone, cartilage, and the bone–cartilage interface with desired biological and mechanical features throughout the regeneration induction process, and to obtain semi-finished or finished osteochondral products that can be directly used for implantation.

In bone regeneration, obtaining an appropriate scaffold and bioactive molecules does not seem to be a problem. However, precisely controlling the release of the bioactive molecules to avoid undesirable side effects requires further investigation.

For tendon–bone interface regeneration, the goal is to obtain a layer of fibrocartilage between the tendon and the bone. However, neither simple structure materials nor complicated scaffolds can be used clinically to achieve this goal.

The increasing incidence of sports medicine injury is posing clinical challenges to surgeons worldwide. Approaches in the field of regenerative sports medicine will present promising options for the structural and functional restoration of the locomotor system. Unlike tissue regeneration in other systems of the body, the to-be-regenerated structure in the locomotor system should be capable of bearing various types of forces during regeneration without exhibiting obvious deformation and should restore the native connection of the regenerated structure to the different surrounding tissues. The future of sports medicine injury repairing scaffolds relies on using optimal components for the structural materials, bionic 3D structures, which simulate natural structures, and long-lasting viability of the regenerated structure with bioactivity.
