# Corrigendum: Advances in regenerative sports medicine research

**DOI:** 10.3389/fbioe.2022.1035342

**Published:** 2022-09-23

**Authors:** Liren Wang, Jia Jiang, Hai Lin, Tonghe Zhu, Jiangyu Cai, Wei Su, Jiebo Chen, Junjie Xu, Yamin Li, Jing Wang, Kai Zhang, Jinzhong Zhao

**Affiliations:** ^1^ Department of Sports Medicine, Shanghai Jiao Tong University Affiliated Sixth People’s Hospital, Shanghai, China; ^2^ Regenerative Sports Medicine and Translational Youth Science and Technology Innovation Workroom, Shanghai Jiao Tong University School of Medicine, Shanghai, China; ^3^ Regenerative Sports Medicine Lab of the Institute of Microsurgery on Extremities, Shanghai Jiao Tong University Affiliated Sixth People’ Hospital, Shanghai, China; ^4^ National Engineering Research Center for Biomaterials, Sichuan University, Chengdu, China; ^5^ School of Chemistry and Chemical Engineering, Shanghai Engineering Research Center of Pharmaceutical Intelligent Equipment, Shanghai Frontiers Science Research Center for Druggability of Cardiovascular Non-coding RNA, Institute for Frontier Medical Technology, Shanghai University of Engineering Science, Shanghai, China

**Keywords:** regenerative medicine, sports meidicne, meniscus, rotator cuff, cartilage, tendon-to-bone, bone, muscle

In the published article, Jia Jiang was erroneously not listed as the co-first author of the paper. The corrected author list appears below:

Liren Wang^1,2,†^, Jia Jiang^1,2,3,†^, Hai Lin^4^, Tonghe Zhu^5^, Jiangyu Cai^1,4^, Wei Su^1^, Jiebo Chen^1^, Junjie Xu^1^, Yamin Li^1^, Jing Wang^1^, Kai Zhang^4,^* and Jinzhong Zhao^1,2,3,^*

^†^These authors contributed equally to this work

The authors apologize for this error and state that this does not change the scientific conclusions of the article in any way. The original article has been updated.

